# Myosins as fundamental components during tumorigenesis: diverse and indispensable

**DOI:** 10.18632/oncotarget.8800

**Published:** 2016-04-19

**Authors:** Yan-Ruide Li, Wan-Xi Yang

**Affiliations:** ^1^ The Sperm Laboratory, College of Life Sciences, Zhejiang University, Hangzhou, China

**Keywords:** myosin, cancer, tumorigenesis, metastasis development, chromomyosin

## Abstract

Myosin is a kind of actin-based motor protein. As the crucial functions of myosin during tumorigenesis have become increasingly apparent, the profile of myosin in the field of cancer research has also been growing. Eighteen distinct classes of myosins have been discovered in the past twenty years and constitute a diverse superfamily. Various myosins share similar structures. They all convert energy from ATP hydrolysis to exert mechanical stress upon interactions with microfilaments. Ongoing research is increasingly suggesting that at least seven kinds of myosins participate in the formation and development of cancer. Myosins play essential roles in cytokinesis failure, chromosomal and centrosomal amplification, multipolar spindle formation and DNA microsatellite instability. These are all prerequisites of tumor formation. Subsequently, myosins activate various processes of tumor invasion and metastasis development including cell migration, adhesion, protrusion formation, loss of cell polarity and suppression of apoptosis. In this review, we summarize the current understanding of the roles of myosins during tumorigenesis and discuss the factors and mechanisms which may regulate myosins in tumor progression. Furthermore, we put forward a completely new concept of “chromomyosin” to demonstrate the pivotal functions of myosins during karyokinesis and how this acts to optimize the functions of the members of the myosin superfamily.

## INTRODUCTION

Carcinogenesis is a multifaceted and complex process involving alterations in genetic or chromosomal stability [1] that disrupt the normal processes of cell growth and apoptosis progression [2] and thus propel the formation of malignant tumors [3]. Mutated genes and instable chromosomes are prone to lead to changes in cell morphology and physiology. Such aspects include cell polarity loss [4], formation of protrusions [5], adhesion alteration [6], evasion of apoptosis [7], and cell invasion and movement [8]. Furthermore, the interactions between the developing tumor cells and the extracellular matrix microenvironment can also have significant impacts upon tumor progression [9]. The detection and the diagnostic and prognostic capabilities of molecular hallmarks in malignancies has recently become a primary focus of oncology research.

The process of tumorigenesis is under the complicated but precise regulation of various factors. The indispensable roles myosins play in this process has only become apparent in recent years. Myosins constitute a diversiform superfamily of actin-dependent molecular motors which, upon the interactions with microfilaments, convert energy from ATP hydrolysis to mechanical stress [10]. Along with the initial focus upon the conventional myosinII of muscle and nonmuscle cells, there have now been at least 18 distinct classes of myosins discovered over the past twenty years [11]. The human genome contains nearly 40 different myosin-related genes, encoding 12 classes of the myosin superfamily [10]. Myosins typically consist of three functional subdomains: the head domain or NH2-terminal motor domain is required for actin binding and ATP hydrolysis and produces the primary aspects of mechanical power [11]; the neck domain, including one or more IQ motifs (consensus sequence IQXXXRGXXXR, where X is any kind of amino acid), acts to efficiently bind calmodulin and light chains [12]; and the tail domain or COOH-terminal domain, is highly divergent and class-specific and participates in cargo transport at along microfilaments. This latter tail region contains a coiled-coil α-helix which promotes the dimerization of heavy chains and the formation of bipolar filaments. Other special properties of the tail region are also responsible for signal transduction and membrane interaction [12].

Previously, we have demonstrated the essential roles of the myosin superfamily in spermatogenesis and in reproductive system disease [11]. Others have also covered aspects such as myosin I's role in in intracellular transport and acrosome biogenesis [13]; myosin II and X's involvement in spindle assembly and karyokinesis [14]; myosin V's association with acrosomal formation and nuclear morphogenesis [15]; myosin VI's correlation with the unequal partitioning of both organelles and cytoskeletal components [16]; and myosin VII's special function in the functional maintenance of Sertoli cells [17]. Furthermore, these six classes of myosins also exhibit functions in reproductive system diseases, such as in testicular tumors and prostate cancer [11]. In recent years, increasing evidence indicates that myosins play multi-functional and crucial roles during tumorigenesis. This has led to a focus upon them as potential therapeutic targets for cancer-related diseases.

The overexpression of myosins has been reported in various cancers, including colorectal cancer [18–21], prostate cancer [22–27], breast cancer [28–30], ovarian cancer [31–32], melanoma [33–34], intestinal neoplasia [35–36], gastric cancer [37–38], pancreatic cancer [39–40], anaplastic gliomas [41] and acute myeloid leukemia [42]. In each cancer type myosins seem to play different but necessary roles during tumorigenesis (Table 1). Myosin IE is a component of the actin-rich core of invadosomes where specialized cell-substrate adhesion and other associated structures display extracellular matrix degradation and tumor cell invasion [43]. Myosin II links to actin filaments and drives cancer cell motility [44]. A depletion of myosin II inhibits tumor invasion and migration [33]. The assembly dynamics of myosin II in its interactions in the process of integrin engagement have also been implicated in cell protrusion formation [45]. Positive regulation of myosin Va by Snail also seems to be integral in the migration of metastatic cancer cells [19]. The overexpression of myosin V facilitates actin assembly and cell motility whilst a deficiency invariably acts to inhibit cell spreading, cell migration and the formation of cytoskeletal architecture. This indicates the pivotal roles of myosin Va in cell motility and cytoskeleton organization [46]. Myosin VI, as an early marker of prostate cancer development [23], not only regulates DNA damage repair in response to the p53 protein (a tumor suppressor protein), but also takes part in the dissemination of ovarian cancer [31], maturation of cadherin-mediated cell adhesion during polarization [47–48] and Golgi apparatus functional maintenance [49]. Myosin VII indirectly associates with the cadherin-catenin complex through a novel transmembrane protein, vezatin, and exerts a tension stress between the actin cytoskeleton and adherens junctions, thereby strengthening cell adhesion and inhibiting tumorigenesis [50]. Myosin IX can down-regulate Rho activity and prevent actin bundle assembly during the nascent formation of cell adhesion [51]. Suppression of Myosin IX affects collective migration and causes cell scattering, two of the most critical factors of cancer cell motility [52]. Myosin X is localized at the tips of filopodia [53] and is active in filopodia formation [54]. In cancer cells, the impairing of p53 can promote increased myosin X expression levels, myosin X being responsible for cell adhesion inhibition, protrusion formation and tumor progression [55]. Various of these and other kinds of myosins and myosin-related molecules participate in other aspects of the formation and development of cancer cells. Further investigation of the many, varied and complex connections and mechanisms that exist between myosins and tumorigenesis will be necessary for future oncotherapy.

**Table 1:** Information of various myosins related to tumorigenesis

Myosin type		Fuctions	Cancer types	Species	Contributors
Myosin I		Recruit invadosome components to plasma membrane, transport vesicles		Hamster	[130]
Myosin II	Myosin II	Cell invasion and adhesion	Pancreatic cancer	Human	[39]
			Breast cancer	Human	[28–29]
			Prostate cancer	Mouse	[24]
			Anaplastic gliomas	Human	[41]
		Regulate tumor cell migration by interacting with P-cadherin	Melanoma	Human	[33]
		Maintain cell polarization, stabilize nascent focal adhesion complexes, mediate efficient integrin-based cell migration	Breast cancer	Human	[112]
		Promote the cell adherens junction and cadherin accumulation in response to E-cadherin,		Human and hamster	[146]
	Myosin IIA	Promote two-dimensional epithelial cell movement but prevent three-dimensional invasion in cancer metastasis		Human	[93]
		Link to actin filaments and take part in cancer cell motility	Breast cancer	Human	[30]
		Regulation of actin retrograde flow		Human and rabbit	[127]
	Myosin IIB	Establish front–back polarity and guide organelle/nuclear orientation		Hamster	[112]
		Participate in cell migration by maintaining protrusion stability		Mouse	[113]
		Downregulate P-cadherin and facilitate tumor cells invasiveness	Melanoma	Human	[33]
Myosin V	Myosin V	Lysosome trafficking and inheritance		*Saccharomyces cerevisiae*	[104]
		Trafficking of apical and basolateral proteins, regulate epithelial cell polarity	Gastric cancer	Human	[38]
	Myosin Va	Migration of metastatic cancer cells and cytoskeletal organization	Colorectal cancer	Human	[19]
		Mediate the functions of Bcl-xL in tumor cell migration and spreading		Mouse	[102]
Myosin VI		DNA damage repair and tumor suppression		Human	[82]
		Dissemination of cancer cells, border cell migration	Ovarian cancer	*Drosophila* (model)	[31]
			Prostate cancer		[25]
		Regulate the maturation of cadherin-mediated cell adhesion during polarization		*Drosophila*	[136]
		Normal material transportation and maintenance of Golgi structure and function	Prostate cancer	Human	[164]
Myosin VII		Interact with cadherin-catenins complex and strengthen cell adhesion		Human and mouse	[50]
Myosin IX		Down-regulate Rho activity and actin bundle assembly, affect collective migration		Human	[52]
Myosin X		Response to impaired p53, cell adhesion inhibition, protrusion formation and tumor progression	Breast cancer	Human	[55]
		Promote filopodia formation, metastasis development	Primary glioblastoma	Human	[133]
			Acute lymphoblastic leukemia	Human	[134]

In summary, cancer-related diseases remain of global importance and the exploration of the correlative molecules and regulatory mechanisms underlying tumorigenesis has become a prime focus of oncology research. Recently, the functions of the myosin superfamily during tumor progression has become of increasing interest. Moreover, some detailed pathways and specific mechanisms related to it have been illustrated. We summarize nine distinctive pathways and myosins related up- or down-stream molecules, including MLCK, PKM2, Sds22, Cdc42, integrin-β1, syndecan-4, merlin, RhoGEF2, Rho GTPases and phytate hydrolysate, etc. Among these factors, the membrane protein integrin-β1 has gradually generated scientists' attention. Integrin-β1 downregulates Cdc42 and TGF-β2 and results in MLCK activation. This activity can accelerate cellular proliferation and tumorigenesis. These pathways and molecules help us better understand the process of tumorigenesis.

In this review, we summarize current studies and demonstrate the integral connections between myosins and cancer. Meanwhile, we provide a prediction of the likely and logical progression of such studies related to the role of myosin in these mechanistic regulatory mechanisms and suggest some possible outcomes that may lead to new or improved medical therapies for cancer. Finally, we put forward a completely new concept of “chromomyosin” to illustrate the special functions of myosins during karyokinesis and perfect the concept of myosin superfamily.

## EFFECTS OF MYOSINS ON GENETIC OR CHROMOSOMAL INSTABILITY

It is well known that carcinogenesis results from the accumulation of gene mutations related to factors controlling the growth of an organism. However, many of the related mechanisms by which such mutations are generated and function remain unknown, despite having been investigated for decades [56]. Accumulating evidence does show that underlying genetic instability is a major cause of gene mutation and acts in the inducement of tumor progression [57]. There are two kinds of such instabilities that can exist in genes. The first of these is nucleotide level instability. This is involved in a small proportion of cancers where which base insertions, deletions and substitutions occur. However, it is chromosome level instability that is the underlining instability for most cancers. This consists of a gain or loss of a whole chromosome, or the gain or loss of a large part of a chromosome. These instabilities are prone to cause elimination of normal tumor suppressor genes and other genetic impairments. These can subsequently promote uncontrolled growth characteristics and reduced survival response to cell damage. These findings provide new insights into tumorigenesis and cancer-related diseases.

Myosins have raised an interest related to tumor progression and cancer therapy because of their effects towards ongenetic and chromosomal instability. Increasing evidence suggests that myosins play essential roles in cases of cytokinesis failure [58], chromosomal and centrosomal amplification [59], multipolar spindle formation [55] and DNA microsatellite instability [36]. Identifying myosins' specific functions and clarifying their related mechanisms have become a prime focus in oncology.

### Chromosomal and centrosomal amplifications

An unstable genome, often related to amplified chromosome or centrosomal numbers, are invariable occurrences in all cancer cells [60]. It is these aspects that are the hallmarks malignant transformation of cells and tumorigenesis [18]. Increasing evidence suggests that these chromosome and centrosome aberrations have been also found in early premalignant lesions. This observation further supports their pivotal roles in tumorigenesis [61]. It is believed that the majority of malignant tumors in humans are associated with chromosomal instability (CIN) involving aneuploid or polyploid karyotypes and structural chromosome aberrations [62]. Underlying genomic or chromosomal instability may stimulate the accumulation of mutations and hence CIN is regarded as the initiation point of tumorigenesis [63]. CIN has been observed in various cancers, including glioblastoma multiforme [64], cervical malignancies [65], colonic adenocarcinoma [66] and other tumor-derived cell lines [67]. In addition to CIN, divisional failures also cause chromosomal and centrosomal amplifications [61]. Cytokinesis failures are prone to produce multipolar mitotic spindles. These lead to uneven chromosome segregation [68]. During cell division, centrosome aberrations also impair the positioning of the cleavage plane and the accuracy of chromosome separation. This results in multipolar spindle formation in the following phase of mitosis [2]. In addition to tumor cells, centrosome amplification and associated multipolar mitoses also takes place in the early stages of inflammation [69]. These studies indicate that the CIN and cytokinesis failures, together with the related chromosomal and centrosomal amplifications, play crucial roles in tumorigenesis and cancer development.

The detailed mechanisms of cell division failures in cancer cells are also related to myosins and their associated molecules [58]. Wu et al. [59] found that cancer cells always failed at cytokinesis because of reduced phosphorylation of the myosin regulatory light chain (MLC). This limited MLC phosphorylation level was correlated with the overexpression of myosin phosphatase (MYPT1) and decreased expression of myosin light chain kinase (MLCK). Conversely, the elevated phosphorylation of MLC markedly inhibited division failures in tested cancer cells. These results demonstrate novel relationships between molecular deficiency and cytokinesis failure and prove the important function of myosin-related proteins in tumorigenesis. Using microarray analyses, it was found that the expression of the MLCK gene was decreased in a variety of tumors, including lung, testis, brain, breast and prostate tumors [70]. The MLCK transcription and expression levels also exhibited dramatic reduction after oncogenic transformation in mesenchymal tumor cells [71] and virus-transformed chicken embryo fibroblasts [72]. Based on the above experimental results, we can put forward a reasonable regulatory pathway associated with myosin-related proteins and cytokinesis failure. At the initial stage of tumorigenesis, the expression of MLCK is inhibited, but that of MYPT1 is elevated. These two proteins then interact with MLC and suppress MLC's phosphorylation level. This leads to cytokinesis failures and facilitates tumor progression. Taken together, these findings indicate MLCK, MYPT1 and MLC as potential agents for future cancer therapy.

### DNA microsatellite instability

DNA microsatellite instability (MSI) is a form of genomic instability related to the impaired DNA mismatch repair (MMR) system in tumors [73]. MSI has become a symbol of particular tumors, including for those related to colorectal cancer [20] and intestinal neoplasia [60]. MMR-deficient cells rapidly accumulate mutations. This leads to gene instability and affects short repetitive DNA sequences. Under such circumstances the sequences containing mononucleotide microsatellites within the coding region typically tend to inactivate and cause MSI-related tumorigenesis [74]. Although most mutations act as “passengers” and do not severely affect cellular morphology and function, there are also some “driver” mutations which directly or indirectly contribute to cancer development and malignant progression.

It was reported that *MYH11*, a smooth muscle myosin gene, was down-expressed in MSI colorectal cancer as compared with levels in normal tissues [21]. There are two splice variants of the *MYH11* gene, SM1 and SM2, and indeed, the SM2 isoform contains a repeated mononucleotide of eight cytosines (C8). This promotes *MYH11* as a candidate gene of MSI-related cancers [60]. Other results have suggested that the mutated *MYH11* is not involved in early tumor formation but participates in the process of MSI tumorigenesis [20]. In addition to the cases of colorectal cancer, smooth muscle myosin-related genes are also implicated in various inherited human diseases such as acute myeloid leukaemia [42], thoracic aortic aneurysm [75–76] and sarcomere and skeletal muscle diseases [35]. The specific mechanisms of the relationship between the *MYH11* gene and myosins in cancer cells requires further investigation.

### p53-dependent regulation

p53 is a tumor suppressor protein which can inhibit tumor progression by acting on a series of p53 target genes. Based on their diverse functions, these genes have been classified into various different categories. P21 is associated with cell cycle arrest; DDb2 and XPB mediate DNA damage and repair; Bax and Fas are involved in cell apoptosis; and VEGF functions in anti-metastasis and anti-angiogenesis [77]. In both mouse and human cells, depletion of p53 always results in cytokinesis failure [78] and spontaneous tetraploid formation [79]. Loss of p53 can also facilitate mutations related to genomic or chromosomal instability [80].

Myosin VI is often considered as a motor protein participating in organelle trafficking and the maintenance of Golgi complex [49]. However, more recently it was found to be also required for DNA damage response [81]. Jung et al. [82] suggested that myosin VI may be regulated by the p53 protein and that DNA damage would occur in a p53-dependent manner. p53 can specifically and directly bind to the myosin VI gene promoter and activate its expression. The intracellular location and functions of myosin VI are subsequently changed responsively in a p53-dependent manner. Moreover, inhibition of myosin VI can impair the integrity of the Golgi complex and suppress the activation of p53. This tends to cause DNA damage and cell apoptosis [82]. The above results demonstrate the interaction between myosin VI and the p53-dependent regulation involved in DNA damage repair and tumor suppression.

A large body of research shows that p53 depletion facilitates tumor cell invasion and metastasis development [83]. One reported mechanism related to mutant p53-induced metastasis is the accelerated accumulation of β1 integrin in the plasma membrane [84]. β1 integrin is a kind of cell adhesion receptor and is involved in filopodia formation and cell invasion [85]. In cancer cells, impaired p53 can promote increased myosin X expression levels, while suppression of endogenous mutant p53 inhibits myosin X expression and its corresponding function in cell migration. The upregulation of myosin X in depleted p53-driven malignancies is implicated in cell adhesion inhibition, protrusion formation and tumor progression [55]. This provides a clinically important invasion mechanism that may provide opportunity for therapeutic intervention.

Allelic loss at 17p, as a most frequent chromosomal deletion, often takes place in human malignancies [86]. Within the same region, some tumor suppressor loci, such as *TP53*, undergo alteration in most tumors. *Tp53* is a typical tumor suppressor gene located at 17p13.3 which plays an important role in the regulation of normal cell morphology and function [87]. In addition, qPCR statistical analysis and genetic screening has indicated that *Myo1c* and *Innp5k* serve as the two other prominent tumor suppressor genes in the *TP53* locus. These two genes are located adjacent to each other and show down-regulation in human tumors [88]. Although the detailed functions of the two candidate genes during tumorigenesis remain unknown, it is reasonable to assume their related proteins, myosin 1c and INNP5K (inositol polyphosphate-5-phosphatase K), participate in tumor suppression or have an anti-metastasis function.

## ROLES OF MYOSINS IN TUMOR INVASION AND METASTASIS DEVELOPMENT

The mechanism of tumor invasion and metastasis development are commonly considered to involve the migration of neoplastic cells that have detached from the primary tumor, entered lymphatic and/or blood vessels and germinated in distant organs [4]. The molecules or other regulatory factors involved in these processes are obviously recognized as new prognostic markers for tumor formation [89]. Metastasis formation is a complex multistep process [90]. It involves invasion through the acquisition of cell motility, the degradation of extracellular matrices and basement membranes, adherence, extravasation and tumor cell proliferation [29]. Fundamentally, these physiological processes are relevant for the movement of any cells from one site to another [91]. Some treatments aiming at inhibiting tumor invasion and metastasis development have become strong candidates for further development and attention and may result in many aspects of therapeutic improvement for many cancer-related diseases.

Myosins, as defined by their roles as actin-based molecular motors that translocate along microfilaments in an ATP-dependent manner, have irreplaceable functions in many aspects of eukaryotic motility such as cell movement, cytokinesis, phagocytosis, organelle/particle trafficking, signal transduction and in the maintaining of cellular morphology [92]. In addition, there are some experimental results indicating that myosins are also required for various other cellular functions related to the formation of cancer. These include cell migration and tumor metastasis. It has been suggested that the blocking of myosin II activity by specific myosin light chain kinase inhibitors could prevent invasion by, and adhesion of, pancreatic cancer [39] and breast cancer cells [29]. Depletion of myosin II, as mediated by P-cadherin, inhibits the invasion by, and migration of, melanoma cells [33]. Myosin II also has multiple functions in the migration of colonic epithelial cells and in a number of aspects of the interplay between F-actin and cell-matrix adhesion. Previous work has demonstrated that myosin IIA can act to promote two-dimensional epithelial cell movement, as occurs in wound closure, but prevent three-dimensional invasion in cases of cancer metastasis [93]. A mutation in *MYH11* (expressing the smooth-muscle myosin heavy chain) results in human colorectal cancer [20] and intestinal neoplasia formation [60]. González et al. [18] showed a positive relationship between myosin expression and tumor recurrence in colorectal cancer patients. This was associated with tumor aggressiveness and metastasis development. Positive regulation of myosin Va by Snail is also implicated in the migration of metastatic cancer cells [19].

In conclusion, based on the above experimental studies, it is suggested that a number of different myosins play various and important roles in tumor invasion and metastasis development. Thereby, it is reasonable to treat myosins as new potential therapeutic targets against tumor formation and carcinogenesis.

### Myosins, cytoskeleton dynamics and cancer cell motility

The development of cancer metastasis is a multistep and complex process, the prerequisite of which is cell motility [94]. The process presumably relies on various activities relating to the cytoskeleton, actin polymerization, cell adhesion and other acto-myosin dynamics [95]. There is increasing evidence indicating that myosins, together with other actin filament and adhesion proteins, play crucial roles during cancer cell migration.

Cancer cell motility and contractile motion are relevant for the continuous structural alternations required for assembly and disassembly of the actomyosin cytoskeleton [96]. Non-muscle myosin II is a major component of actomyosin bundles [97]. Increasing evidence suggests that myosin II proteins, especially the myosin IIA isoform, link to actin filaments and take part in cancer cell motility [44]. MYBPH (myosin binding protein H) directly interplays with NMHC IIA (non-muscle myosin heavy chain IIA) and indirectly inhibits myosin IIA assembly. This leads to a reduction of cell motility [98]. Interestingly, when treated with ROCK inhibitor, myosin IIA is altered from a disassembly state to an assembly-competent state where it then suppresses the interaction between MYBPH and NMHC IIA. This indicates a higher affinity between MYBPH and assembly-competent myosin IIA. The suggestion that myosin II and its related regulatory molecules improve the cell migration and invasion stages of prostate cancer [24], breast cancer [28], pancreatic cancer [39], anaplastic gliomas [41] and melanoma [33] is becoming increasingly apparent. Myosin II may serve as a new therapeutic target for future strategies targeting the inhibition of tumor cell invasion [99–100].

The overexpression of the full-length myosin Va induces actin bundles and cell motility, but reduces the rate of initial cell spreading after plating, whilst abnormal myosin Va, such as that with a tailless structure, disrupts cells from spreading or migrating, and interferes with cytoskeletal architecture [101]. These suggest the essential roles of myosin Va in cell motility and cytoskeleton organization [46]. Lan et al. [19] found that the mRNA expression of myosin Va was highly increased in metastatic colorectal cancer cells. Suppressing myosin Va expression inhibited cell migration and metastasis capabilities. Snail, as a transcriptional repressor triggering epithelial mesenchymal transition, can bind to the E-box of the myosin Va promoter and positively promote its expression and activity. It has been demonstrated experimentally that myosin Va is required for cancer cell metastasis by affecting cell motility. This provides a novel target for Snail in its regulation of cancer migration [19].

Bcl-xL, a tumor progression factor, is involved in altering the actin cytoskeleton and promoting cancer invasiveness. Myosin Va is identified as an Bcl-xL interplaying regulatory protein which mediates the functions of Bcl-xL in tumor cell migration and spreading [102].

The mechanisms by which myosin V associates with cell motility are still controversial. It is possible that myosin V also participates in cell cytoskeletal reorganization and vesicle/organelle transportation [103]. On the one hand, in *Saccharomyces cerevisiae*, myosin V takes part in the movement of the vacuole/lysosome and is involved in this organelle's inheritance during cell division. Myosin V has a unique tail structure which contains a globular carboxyl terminal domain. This specific tail is thought to regulate localization of myosin and myosin-cargo interplay by special receptors. A mutation at the tail structure of myosin V damages vacuole inheritance and prevents vacuole movement to yeast buds. Immunofluorescence shows that myosin V colocalizes with the vacuole and helps vacuole recruit its membrane to move and become the isolated structure [104]. On the other hand, cancer progression and metastasis are related to altered vacuole/lysosome trafficking and increasing expression of cathepsin, a kind of lysosomal protease [105]. Tumorigenesis triggers a series of alterations in lysosomes, including increasing the permeability of lysosomal membrane and promoting the release of cathepsins into the cytosol. These cathepsins, in turn, stimulate the generation of cell death pathways, such as p53 protein activation, growth factor deprivation and oxidative stress, etc [106–107]. Meanwhile, the cathepsins can efficiently activate caspase- and mitochondrion-independent cell programmed death, resulting in cancer cells death. As a result, we can conclude that myosin V promotes both lysosome trafficking and inheritance in cancer cells, and consequently these alterations trigger cell death pathways and allow cancer cell programmed death.

### Loss of cell polarity and tissue disorganization

Cell polarity is essential for normal cellular function and physiological activity. When mammalian epithelial tumors lose cell polarity, this tends to alter their malignancy [8]. The formation of gastrointestinal tumors are always related to the abnormal cell polarity. Some growth factors, such as EGF, are synthesized in salivary glands and exist in the apical cell surface. While the EGF receptors are limited to the basolateral surfaces of non-transformed cells, so that the growth factors and their receptors interact productively only in the case of local wounding. Disruption of the adhesion junctions after loss of E-cadherin or mislocalization of the growth factor receptors to the apical surfaces of transformed cells lead to increased cell growth and division. Hence, the apical-basolateral cell polarity stimulates gastrointestinal tumorigenesis [108]. Loss of cell polarity, tissue disorganization and invasive cell growth are certainly common observations in most cancers. These affect cell adhesions, asymmetric cell division and metastasis development. Fundamentally, apical-basal polarity has two functions related to tumor suppression in epithelial cells. These are the regulation of the cell's asymmetric division and the maintenance of the stability of the apical junction complex [109].

Recently, it was found that a deficiency of myosin V could disrupt the trafficking of apical and basolateral proteins and inhibit epithelial cell polarity in gastric cancer [138] and in other microvillus inclusion diseases [110–111]. These regulation relationships may be related to Rab and TfR proteins. In migrating cells, myosin IIB is implicated in establishing a front-back polarity and guiding organelle/nuclear orientation [112]. The localization of MTOC (the microtubule-organizing centre), centrosome, Golgi and nucleus are all the remarkable hallmarks of cell polarization. These organelles reorient near the nucleus in the direction of the protrusion [51]. The depletion of myosin II in migrating cells causes multidirectional protrusions [113] and disordered positions of MTOC, Golgi and nucleus [112], resulting in incorrect front-back polarity and impaired cell motility. Increasing evidence suggests that that myosin II serves as a master regulator of cell migration, participating in cell polarization, protrusion formation, adhesion and signaling transduction [114].

### Formation of cell protrusions and cancer cell migration

To migrate and invade during cancer metastasis, the cell body needs to modify its shape and adhesion properties to interact with the extracellular matrix (ECM) and surrounding cells. The ECM microenvironment plays a crucial role in tumor cell progression. It provides a signal substrate and serves as an obstacle to the migrating cell body [9]. Cell migration involves a series of interdependent steps [5], of which the first step is the cell becoming polarized and elongating to form protrusions. Cell protrusions extend from the cells' leading edge and attach to the ECM substrate [115]. Then the leading edge of the cell contracts and generates a traction force. This promotes the trailing edge to glide gradually forward [96]. This process results in various protrusions being formed including filopodia, pseudopodia, invadopodia and lamellipodia [116], which all contain actin filaments and other motor proteins dynamically interacting with ECM substrates. Cell extension and protrusion formation are the prerequisites for cancer cell progression [4].

Initial cell elongation and protrusion formation is driven by the actin filament assembly [117]. Subsequently, integrins interplay with actin filaments and link the contractile force of actomyosin to the extracellular environment [118]. This clusters cytoskeletal components which are then able to develop into focal contact [119]. These processes are facilitated by the action of crosslinking proteins including myosin II and α-actinin [117] and regulatory molecules involving MLCK, small G-protein RHO, ROCK (RHO-associated serine/threonine kinase)[120], FAK (focal adhesion kinase) [121] and MMPs (matrix metalloproteinases) [122].

During the formation of the leading-edge of cell protrusions, cell migration is driven by the force of actin-network expansion [95] and supported by a squeezing *via* myosin II-dependent contraction which is able to propel the rigid nucleus through narrow spaces [118]. Assembly of myosin II into the cell cortex is involved in stabilizing nascent focal adhesion complexes and mediating efficient integrin-based cell migration [114]. These are the crucial processes in the control of cell motility, adhesion and tissue architecture [123]. Proteomic analysis has suggested that in breast cancer cells, MHC (myosin heavy chain) phosphorylation sites were activated during integrin engagement and lamellar extension on fibronectin [30]. Although myosin II is not indispensable for the nascent formation of lamellipodium, it is truly essential to the subsequent cell's elongation and growth [124–125]. The assembly dynamics of myosin II's interaction with integrin engagement plays important roles in regulating the components of cancer cell migration during tissue setting [45]. Further studies are required to identify the factors and mechanisms involved in this process as well as those related to the invasion strategy of cancer cells beyond any pharmacotherapeutic intervention.

In fibroblasts, two isoforms of myosin II, myosin IIA and B, have been described. As suggested by their differing subcellular localizations, ATPase activities and contraction capabilities, they may play different roles in the regulation of the actin cytoskeleton [126]. Myosin IIB participates in cell migration by maintaining protrusion stability [113]. Myosin IIA is required for the regulation of actin retrograde flow [127]. Studies have suggested that the depletion either myosin isoforms could propel cell protrusion and prevent nascent cell adhesion [112]. Myosin IIA and myosin IIB both exist external to protrusions and promote cell elongation, signaling and the maturation of adhesions, at a distance [128]. Myosin IIA controls adhesion dynamics and myosin IIB exerts front-back polarity and substrate orientation functions [129].

Myosin I E (myo1e) is a component of the actin-rich core in the invadosome. The invadosme is a specialized structure of cell-substrate attachment which associates with ECM degradation and invasion [43]. During invadosome expansion, myo1e serves as a scaffold to recruit invadosome components to the plasma membrane and transport vesicles to the sites of new invadosome assembly. Inhibition of myo1e contributes to mislocalized invadosomes at the rosette center whereas normal myo1e expression is implicated in new invadosome formation at the periphery of the rosette [130].

Myosin X is the best known for its localization to the tips of filopodia [53] and its role in filopodia formation [54]. Myosin X overexpression promotes the formation of hundreds of filopodia in one cell and its knockdown inhibits endogenous filopodia [131]. Increasing experimental data shows that myosin X is implicated in cancer cell's protrusions and metastasis development by transporting β1 integrins to the filopodia tips [132]. High expression levels of myosin X are also observed in primary glioblastomas [133], acute lymphoblastic leukemia [134] and pathogen infections [55].

The formation of cell extension and protrusion are the prerequisites for cancer cell migration, and understanding the process of myosins stimulating cancer cells' movement in tissues or organs is of vital importance. Recently, myosin VI was found to have crucial functions in the dissemination of ovarian cancer. During the normal development process of the *Drosophila* ovary, there is a dynamic stage known as border cell migration. This stage shares a series of similarities at molecular and behavioral levels with human ovarian cancer invasion [32]. Border cell migration in the *Drosophila* ovary is a process in which a partial epithelial cells gradually turn into mesenchymal cells and invade the neighboring germline cells. A key gene that regulates this transition activity is *slbo* (slow border cells), which encodes *Drosophila* C/EBP protein. C/EBP is a transcriptional activator and improves the expression content of the downstream targets, including FAK (focal adhesion kinase) and E-cadherin [135]. These factors function together in promoting cells migration and motility. Throughout ovary border cell migration, myosin VI is highly expressed in these border cells. High magnification confocal micrographs suggest that border cell myosin VI expression is specifically decreased in *slbo* mutant type compared with the wild type. Meanwhile, myosin VI interacts with E-cadherin and Armadillo (Drosophila β-catenin) to control cell-cell adhesion and migration. The expression of E-cadherin and Armadillo dramatically reduced in myosin VI deficient cells; myosin VI is inhibited in the cells lacking Armadillo or E-cadherin [136].

Initially, the polarized epithelial cells form specific cell-cell junctions which contain distinct membrane subdomains and adhesion molecules. When these cells gradually migration and turns into mesenchymal cells, the cell polarity inverts from apical to planar, and the adhesion proteins located in the cell-cell junctions reorganize at the protrusion of the border cells. Meanwhile, myosin VI translocates from the junctions to the membrane edge at the invasive front of the cells, thus accelerating cell migration and causing tumor invasion [23]. Emerging experimental evidence suggests myosin VI regulates border cell migration and is highly expressed in metastatic ovarian cancer but not in normal ovarian tissue. Suppressing myosin VI expression prevents cancer cell invasion and migration [31]. The border cell migration mediated by myosin VI has served as a useful paradigm for cancer cell invasion [137]. Another similar relationship between myosin VI and the spreading of cancer cells has also been observed in prostate cancer [25], where the analysis of the detailed mechanisms suggest possible myosin VI involvement in the maintenance of cell polarity, cell-cell adhesion and material transportation [48].

### Cadherin-mediated cell adhesion

EMT, epithelial-to-mesenchymal transition, is a physiological process where epithelial cells lose polarity, impair cell-cell adhesion and become more invasive. EMT occurs in a variety of cell or tissue developmental processes, especially those related to tumor progression [6]. During EMT, the actin cytoskeleton undergoes structural changes from a circumferential band of filaments to stress fibers where a number of related proteins and molecules play essential roles [138]. The cell adherens junction functions in regulating EMT and correlative activities, where cadherins are the major adhesion molecules in the adherens junction.

Cadherin is calcium-dependent cell adhesion molecule. It strengthens the cell-cell junction through having repeated extracellular domains and linking to the actin cytoskeleton *via* its cytosolic domain connection to catenins [138]. The cadherin family consists of E (epithelial)-, N (neuronal)-, P (placental)-, and VE (vascular endothelial)-cadherins, named after the tissues within which they were first found. Cadherins participate in the establishment of cell polarity, cell invasion and the formation of metastasis [139]. E-cadherin is expressed in normal epithelial tissues but has lost its expression in epithelial derived cancer cells [140]. In cancer cells, downregulated E-cadherins always contribute to a decreased cell-cell adhesion, thus increasing the invasive and metastatic capabilities of cancer cells [141]. However, it is interesting that epithelial derived cancer overexpresses N-cadherin and that the upregulation of N-cadherins has the similar function to the downregulation of E-cadherin [142]. At the initial stage of tumorigenesis, cells undergo a series of physiological changes including abnormal alternation of cadherin types and quantities, a process known as cadherin switching. This process involves a switch from the expression of E-cadherin to N-cadherin [143]. Cadherins play important roles in cell adhesion, migration and cancer metastasis, and defects in cadherin function and expression are characteristic of many cancers.

**Figure 1:**
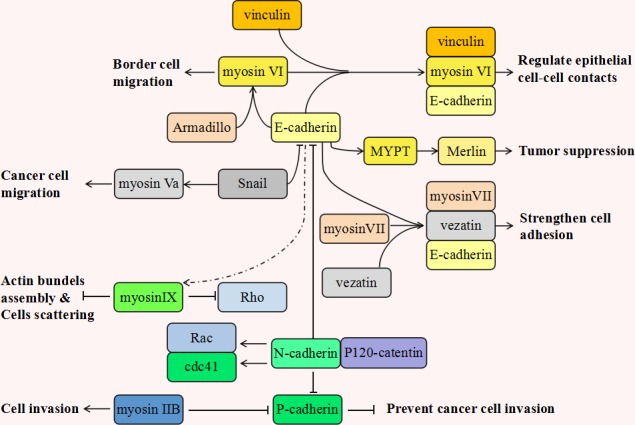
The inseparable relationship between various myosins and cadherins during tumorigenesis

Cadherins link to the actin cytoskeleton through the cytosolic domain and interact with many motor proteins including myosins, kinesins and dyneins (See Figure 1) [144]. In mammalian epithelial cells, myosin VI promotes the maturation of cadherin-mediated cell adhesion during polarization [48]. Myosin VI forms a strong biochemical complex with E-cadherin. This enables epithelial cells to form linear cohesive contacts and facilitates the integrity of the apical junctional complex. In addition to E-cadherin, vinculin also regulates the effects of myosin VI. It is these three proteins functioning together, (myosin VI, E-cadherin and vinculin), that critically mediate the morphogenesis of epithelial cell-cell contacts [145]. In *Drosophila melanogaster*, the protein levels of E-cadherin and Armadillo (*Drosophila* β-catenin) are dramatically reduced in the cells lacking myosin VI. Similarly, myosin VI is inhibited in the cells lacking E-cadherin and Armadillo. Data suggests that myosin VI is involved in border cell migrations where it stabilizes and interplays with E-cadherin and Armadillo [136]. In epithelial cells, myosin II also can be recruited and activated in response to E-cadherin, where it functions in the promotion of the cell adherens junction and cadherin accumulation [146]. Myosin Va is activated by Snail to facilitate cancer cell migration [19]. Concurrent to this, Snail has been shown to suppress normal epithelial cell adhesion by down-regulating the expression of genes encoding adherens proteins such as E-cadherin, claudins and occludin [147]. Myosin VII indirectly associates with the cadherin-catenins complex through a novel transmembrane protein, vezatin, which is a ubiquitous protein of adherens cell-cell junctions. Myosin VII is anchored by vezatin, along side the cadherin-catenins complex. It exerts a tension force between the actin cytoskeleton and cell adherens junction, resulting in a strengthening of cell adhesion [50]. Myosin IX locally down-regulates Rho activity and the actin bundle assembly during the nascent formation of cell adhesions [148]. Depletion of Myosin IX affects collective migration and causes cellular scattering, both critical factors of cancer cell motility [52].

This figure shows that the different classes of myosins and cadherins play multiple and even antagonistic roles during tumor progression. Myosin VI forms a strong biochemical complex with E-cadherin and vinculin, which critically regulates the morphogenesis of epithelial cell-cell contacts. In *Drosophila melanogaster*, the protein levels of E-cadherin and Armadillo (*Drosophila* β-catenin) have positive correlations with that of myosin VI. Myosin VI takes part in border cell migration and can stabilize and interplay with E-cadherin and Armadillois. In epithelial cells, myosin II also can be recruited and activated in response to E-cadherin where it functions in promoting cell adherens junction and cadherin accumulation. Myosin Va is activated by Snail to promote cancer cell migration. Concurrent to this, snail has been shown to suppress normal epithelial cell adhesion *via* the down-regulation of the expression of adherens proteins such as E-cadherin, claudins and occludin. Myosin VII links to vezatin and E-cadherin and forms a cadherin-catenins complex exerting a tension force between the actin cytoskeleton and cell adherens junctions, thus strengthening cell adhesion. Myosin IX locally down-regulates Rho activity and the actin bundle assembly during nascent formation of cell adhesions. It is reasonable to assume that E-cadherin can functionally activate myosin IX and inhibit cell scattering. E-cadherin also can activate MYPT and this leads to a “closed” active merlin, resulting in tumor suppression. Among the various cadherins, cadherin switching also takes place. This means N- cadherin gradually takes the place of other cadherins including E- cadherin and P- cadherin. Cadherin switching may be induced by P120-catenin which is involved in cadherin's endocytosis. This results from the different cadherins competing for the binding to p120-catenin. In contrast to the actions of E- and P-cadherin, N-cadherin can maintain steady levels of phosphorylated Rac and Cdc42 thus improving cell motility and aggressiveness. It is reported that P-cadherin is downregulated in metastasizing melanomas and over-expression of P-cadherin can efficiently prevent cancer cell invasion. This process is corrected with the inhibition of myosin IIB, which contributes to tumor cells invasiveness by retracting the tail during the migratory cycle.

In human melanoma progression, the malignant melanocyte's transformation is frequently associated with cadherin switching from E- to N- [149]. P-cadherin has also been noted to be downregulated in metastasizing melanomas and the over-expression of P-cadherin could efficiently prevent cancer cell invasion. Great progress has been made in the identification of the migration-related molecular mechanisms underlying the cutaneous melanoma metastatic process. Non-muscle myosin IIB has been discovered to downregulate P-cadherin in melanomas and myosin IIB contributes to melanoma invasiveness by triggering tail retraction during the migratory cycle [33]. The inverse relationship between myosin IIB and P-cadherin may be significant and strategic to the melanoma cell's deeper migration into the stroma in metastasis.

Many papers have suggested that myosins function with cadherins to enhance cell adhesion and migration. Some myosins form adhesion complexes with the underlying cadherin substrates to strengthen and stabilize cell junctions. Others inter-regulate with cadherins and other membrane proteins to exert other influences. Although ample evidence supports the roles of myosins in cell adhesion and cancer invasion, detailed studies are still required to elucidate the related specific molecular mechanisms and to elucidate the other interacting proteins involved in myosin-related cancer cell migration and metastasis development.

### Suppression of apoptosis

Apoptosis, a kind of programmed cell death in vertebrates, involves a series of morphological feature changes at a cellular level. These include nuclear/cytoplasmic fragmentation, cell shrinkage, formation of dense bodies and global mRNA decay [28]. Previous studies have suggested that crucial apoptotic modulators have been deregulated or inhibited in metastatic cancer cells and that the suppression of apoptosis promotes tumor invasion and metastasis development [150]. Deficient regulation of apoptosis is a general occurrence in human malignances. Cancer cells may acquire their aggressive capabilities because of these apoptosis deficiencies. The altering of cancer cells into an apoptotic status by anti-neoplastic agents has now become a major therapeutic method [151].

The high metastasis potential breast cancer, LM-MCF-7, cells display stronger anti-apoptosis abilities than normal breast cancer MCF-7 cells. This is attributed to both the upregulation of MLCK, myosin light chain kinase, and the downregulation of p-p38 (phosphorylated p38) in the LM-MCF-7 cells. Suppression of MLCK, as mediated by ML-7, could induce apoptosis in the LM-MCF-7 cells and increase the level of p-p38 [28]. Potentially, MLCK is likely to impact tumor invasion and metastasis development by mediating apoptosis and may thus serve as a novel therapeutic target for breast cancer [152].

### Functional maintenance of Golgi apparatus

The Golgi apparatus, the indispensable subcellular structure primarily involved in modification, sorting and packaging of macromolecules, has been recently found to play an essential role in carcinogenesis [153]. When cells enter mitosis, the pericentriolar Golgi apparatus is regulated by mitotic signals and proceeds to fragment and disperse [154]. In this process, the Golgi apparatus mediates a series of proteins and molecules which are crucial for cell signaling, transportation and apoptosis. In addition, the reorientation of the MTOC, Golgi apparatus, and nucleus are symbols of cell polarization [155]. Studies are increasingly elucidating the important functions of Golgi in cell polarity, migration, apoptosis and mitotic spindle formation. All of these are all the necessary requirements of cancer cell invasion and metastasis development.

The interactions between myosins and Golgi apparatus always attract people's attention. Myosin I functions in post-Golgi apparatus transportation, endocytosis and exocytosis, membrane trafficking and endo-lysosomal traffic [11]. Microtubules are the transportation sites of the trans-Golgi and they are required for transcytosis of Golgi vesicles from the basolateral membrane to the apical cytoplasm. Throughout the transport activity, myosin I and dynein interact with Golgi membranes, and cross-link Golgi to the actin filaments and the microtubules. An interesting phenomenon is that myosin I locates on all membranes in Golgi apparatus, however, dynein is found only on some small membrane fractions. The motor protein dynein promotes Golgi vesicle to translocate along microtubules to the cell cortex, and then myosin I delivers Golgi to the apical membrane along the microfilaments [13]. In addition, myosin V stimulates post-Golgi vesicle trafficking in the exocytic pathway [13]. Knock-out of myosin V gene arrests cells in an unbudded but enlarged state, with accumulation of secretory Golgi vesicles. These vesicles are prevented from transporting to the bud site and exert normal physiological functions [156]. In mammalian cells, myosin VI is responsible for promoting membrane trafficking pathways. Myosin VI is diffusely localized to endocytic and exocytic vesicle membrane, especially in Golgi apparatus [157], and myosin VI plays a series of vital roles, including maintaining steady organization of the Golgi apparatus [158], post-Golgi vesicle transportation [159] and allowing the delivery of endocytose cargoes to the recycling compartment [160].

GOLPH2 (Golgi phosphoprotein 2) and myosin VI are two candidate Golgi-associated proteins. GOLPH2 is a type-II Golgi membrane protein and serves as a serum marker for various cancers, including hepatocellular carcinomas [161] and prostate cancer [26]. Myosin VI localizes in the Golgi membranes as a peripheral membrane protein and is implicated in the normal material transportation and maintenance of the Golgi's structure and functions [162–163]. In the fibroblasts of Snell's waltzer mice, the absence of myosin VI causes a reduced size of the Golgi and a limited level of protein secretion [49]. Recently, it was found that both GOLPH2 and myosin VI were overexpressed in prostate cancer, where immunofluorescence showed a more obvious Golgi staining of these two proteins in cancer cells than in the adjoining normal epithelium [164]. The increasing expression level of GOLPH2 and myosin VI indicates Golgi-specific alterations related to the molecular composition in cancer cells. These alterations may aid in the deciphering of the detailed mechanisms associated with Golgi apparatus functional maintenance and cancer metastasis development. They could be targeted as novel cancer biomarkers for future therapies.

## MYOSIN-MEDIATED FACTORS, PATHWAYS AND MODELS DURING TUMORIGENESIS

### Myosin-related molecules during tumor progression

**Figure 2:**
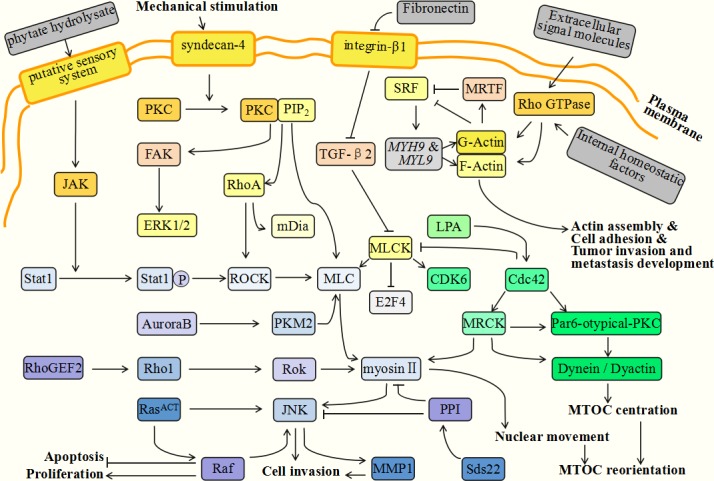
Myosin-related molecules during tumor progression During tumorigenesis, various molecules and factors interact and perform different functions. Among them, myosins and their related up- or down-stream molecules, play irreplaceable roles in the regulation of tumor genetic stability and tumor metastasis development. The extracellular phytate hydrolysate takes part in the formation of a circumferential F-actin ring in colorectal cancer cells. It can stimulate the putative sensory system located in cell membranes and then activate JAK (Janus kinase) and Sata 1. Under the assistance of PKC and JNK, Sata1 induces ROCK (Rho-associated kinase) active and phosphorylates MLC. MLC and the myosin proteins are located at cell-cell contact points and the myosin contractility is responsible for actin assembly and the formation of the circumferential F-actin ring. Syndecan-4 (SDC4) is a member of HSPGs family and is over-expressed in malignant tumor cells. It can efficiently activate PKC to bind with PIP2 (phosphatidylinositol4,5-bisphosphate), and then facilitate the expression of RhoA and FAK. Activated FAK can subsequently phosphorylate ERK1/2, which is required for cell contractility and adhesion. RhoA can activate ROCK and mDia. ROCK is implicated in inhibiting MLC phosphatase and elevating MLC phosphorylation, thus promoting cell contractility and adhesion. The upregulation of integrin-β1 is induced by decreased fibronectin which can facilitate the cell's invasion and growth. Integrin-β1 can downregulate Cdc42 and TGF-β2 and result in MLCK activation. This activity can inhibit growth suppressors, such as E2F4 (E2F transcription factor 4), and activate the cell cycle regulators, such as CDK6 (cyclin-dependent kinase 6). Rho GTPases can be activated in response to extracellular signaling molecules or internal homeostatic factors and function in cytoplasmic effectors which subsequently guide F- and G-actin assembly. The absence of G-actin can inhibit MRTFs, consequently activating SRF and its target genes, such as *MYH9* and *MYL9*. These genes can reversely elevate the activity of actins and contribute to actin assembly, cell adhesion and tumor invasion. During cytokinesis, PKM2 is activated by Aurora B and interacts with MLC2 in the contractile ring region of mitotic cells. RhoGEF2 successively activates Rho1, Rok (Rho kinase) and myosin II. Rok and myosin II then promote JNK (Jun kinase) activation together with *Ras*. JNK is responsible for the inhibition of cell differentiation and tissue growth, and acts to promote cell invasion. Myosin facilitates cell contractility, cytoskeleton reorganization and cytokinesis. These molecules interact together to regulate cooperative tumorigenesis. Sds22 is a regulatory upstream molecule of protein phosphatase 1 (PP1) which is related to the completion of mitosis. PP1 can efficiently inhibit the phosphorylation of myosin II and JNK, thus restricting tumor progression. During cell invasion, MTOC promotes reorientation and facilitates directional movements. The process of MTOC reorientation is modulated by a series of molecules involving Cdc42, myosin II, dynein and PKC. Cdc42 is a small GTPase responsible for activatePar6-atypical-PKC and MRCK (myotonic dystrophy kinase-related Cdc42 binding kinase). Subsequently, Par6-atypical-PKC activates dynein and dynactin and MRCK contributes to myosin II phosphorylation and nuclear movements.

During tumorigenesis, a variety of molecules and factors interact and perform different functions. Among them, myosin proteins and their related up- or down-stream molecules play irreplaceable roles in the regulation of genetic stability and the development of tumor metastasis. Myosins have therefore begun to be treated as new therapeutic candidates for cancer intervention. In the following sections we will list a series of detailed regulatory pathways and reveal the internal relationships between myosins and cancer formation (as summarized in Figure 2).

RhoGEF2, a RhoGTP exchange factor, is an activator of Rho-family GTPases [165]. *Ras* is a typical oncogene which is implicated in about 30% of cancers. However, this gene is not enough to facilitate tumor progression. Recently it was found that RhoGEF2 could cooperate with activated *Ras* (*Ras^ACT^*) or an activated allele of the *Ras* downstream effector, *Raf*, and then affect normal cellular functions. This interaction may be a key initiating factor of tumorigenesis. In this regulatory pathway, RhoGEF2 successively activates Rho1, Rok (Rho kinase) and myosin II after cooperating with *Ras^ACT^* or *Raf*. Rok and myosin II then promote JNK (Jun kinase) activation together with *Ras^ACT^* [166]. JNK can inhibit cell differentiation and tissue growth and promote cell invasion. Myosin II is required for cell contractility, cytoskeleton reorganization and cytokinesis [167]. The above molecules interplay together to accelerate cooperative tumorigenesis.

Sds22 is a regulatory upstream molecule of Protein Phosphatase 1 (PP1), which is relevant for the completion of mitosis [168] and the regulation of epithelial polarity [169]. These two processes are both crucial aspects of malignant tumor regulation. The expression level of PP1 is invariably decreased in cancer cells and the PP1 inhibitor, okadaic acid, can promote tumor invasion and metastasis development [170]. The reasonable interpretation is that PP1 can efficiently inhibit the phosphorylation of myosin II and JNK, thus restricting tumor progression [167]. Furthermore, JNK partly mediates the expression of MMP1 (Matrix metalloprotease 1), which can facilitate cell motility [171] and the degradation of the basement membrane [172], which are both related to tumor malignancy potential [173].

Recently, it was found that phytate hydrolysate could contribute to the formation of circumferential F-actin rings in colorectal cancer cells [174]. Phytate has various biological functions involving apoptosis induction and anticancer activities [175]. It is possible that it is the hydrolysis products of phytate that may exert an anticancer function. The functions of phytate hydrolysate in F-actin ring formation is associated with the JAK-Sata1-Rock-MLC regulatory pathway [174]. Extracellular phytate hydrolysate can stimulate the putative sensory system located in the cell membranes and then activate JAK (Janus kinase) and Sata 1. Under the activation of PKC and JNK, Sata1 activates ROCK (Rho-associated kinase) and phosphorylates MLC. MLC and myosin proteins are located at cell-cell contact points and myosin contractility is responsible for actin assembly and the formation of the circumferential F-actin ring [176]. This pathway provides specific connections between myosins, phytate and corresponding anticancer functions.

HSPG (heparan sulfate proteoglycan) is a kind of polysaccharide located in the cell membrane. It participates in cell adhesion, migration and related physiological processes [177]. Syndecans are the members of the HSPG family and are overexpressed in malignant tumor cells [178]. They are considered to be contributors to tumor progression [179]. Among multiple syndecans, syndecan-4 (SDC4) can efficiently activate PKC to bind with PIP2 (phosphatidylinositol 4,5-bisphosphate) [180]. In addition, under mechanical stimulation, SDC4 also facilitates the expression of RhoA and FAK and acts as a mechanotransducer for the assembly of the actin cytoskeleton, cell contractility and motility [181]. Mechanical stress promotes RhoA activity and then activates its downstream molecules, ROCK and mDia. ROCK is implicated in inhibiting MLC phosphatase and elevating MLC phosphorylation and thus promoting cell contractility and adhesion [182]. Phosphorylated FAK can subsequently activate ERK1/2 and plays a similar role in the activation of MLC [9].

Furthermore, it has been reported that MLCK acts downstream of integrin-β1 and TGF-β2 to promote cell proliferation [27]. In prostate cancer, upregulation of integrin-β1 is induced by decreased fibronectin and can facilitate cellular invasion and growth. The activation of integrin-β1 downregulates Cdc42 and TGF-β2 and results in MLCK activation. This activity can inhibit growth suppressors, such as E2F4 (E2F transcription factor 4), and activate cell cycle regulators, such as CDK6 (cyclin-dependent kinase 6). These factors function together to accelerate cellular proliferation and tumorigenesis [27]. This indicates that TGF-β2-related mechanisms may hold potential to prevent cancers metastasis.

Pyruvate kinase M2 (PKM2) has a high expression level during tumor progression [183] and takes part in spindle assembly and chromosome segregation [184]. Its detailed mechanism is related to MLC2 two-site phosphorylation. During cytokinesis, PKM2 is activated by Aurora B and interacts with MLC2 in the contractile ring region of mitotic cells. Active MLC2 subsequently binds with ROCK2. This leads to ROCK2-dependent MLC2 phosphorylation at two sites of Y118 and S15 [183]. The activated MLC2 then contributes to the progression of cytokinesis for tumor cells [185].

The MTOC, (microtubule-organizing center), is a structure located between the nucleus and the leading edge in migrating cells [148]. During cell invasion, MTOC influences reorientation and facilitates directional movement [186]. The process of MTOC reorientation is modulated by a series of molecules involving Cdc42, myosin II, dynein and PKC [187]. Cdc42 is a small GTPase implicated in Golgi apparatus transportation and MTOC reorientation [188] which can activate Par6-atypical-PKC and MRCK (myotonic dystrophy kinase-related Cdc42 binding kinase). Subsequently, Par6-atypical-PKC activates dynein and dynactin, and MRCK contributes to myosin II phosphorylation and nuclear movements [187]. Taken together, these factors play essential roles in MTOC reorientation and cell migration.

Rho GTPases are implicated in the regulation dynamics of the cytoskeleton *via* multiple effectors which participate in actin-based structural assembly, cell adhesion and migration [189]. It has been recently reported that MRTFs (myocardin-related transcription factors) and SRF (serum response factor) are the downstream factors of Rho GTPases and required for cytoskeletal dynamics and tumor metastasis [190]. Rho GTPases are activated in response to extracellular signalling molecules or internal homeostatic factors and function as cytoplasmic effectors which subsequently guide F- and G-actin assembly [191]. Among them, the absence of G-actin can inhibit MRTFs, consequently activating SRF and its target genes such as *MYH9* and *MYL9*. These target genes encode actin [192] and other cytoskeletal components including NMHCIIa and MLC2. These actin-based factors cooperatively function in cell adhesion, cell spreading and cytoskeleton contractility, thus regulating tumor invasion and metastasis development [193].

Vestibular schwannoma (VS) is a kind of benign tumor which is derived from the internal auditory canal and the eighth cranial nerve. It is associated with hearing loss and even brainstem compression [194]. One cause of VS formation is the inactivation of the *NF2* gene and its downstream molecule, merlin [195]. Merlin shares similarities to the two ERM proteins, moesin and radixin, which both participate in the connection of the actin cytoskeleton to plasma membranes [196]. Merlin has two different states: the “open” inactive state and the “closed” active state. After dephosphorylation at amino acid serine 518, merlin can be folded and activated to act as a tumor suppressor. Signals are transduced into cells by membrane-bound integrins and RTK (receptor tyrosine kinases) which then activate Rac and PAK (p21-activated kinase) [197]. Activated PAK can phosphorylate merlin and induce the “open” inactive conformation [198], thus impairing merlin's functions of tumor suppression. Additionally, merlin also can be phosphorylated by PKA (protein kinase A), which acts not only on serine 518, but also on serine 10. Conversely, cadherin suppresses Pak activation and promotes “closed” active merlin. This similar regulatory pathway is a CD44-related mechanism. CD44 is a transmembrane receptor and anchored to the hyaluronic acid rich matrix. It plays an essential role in cell adhesion and motility [199]. CD44 can activate MYPT1 (myosin phosphatase targeting subunit 1), which acts in opposition to the functions of CPI-17 (protein kinase C-potentiated phosphatase inhibitor of 17 kDa) [200] The activated MYPT1 then leads to a “closed” active merlin, resulting in tumor suppression. The mutual transformation of these two merlin conformations is required for tumor formation or suppression.

Although a series of regulatory mechanisms have been investigated and illustrated, more candidate myosins and related molecules involved in tumorigenesis require elucidation. The interactions between all of these these factors also need to be clarified. How to utilize known mechanisms to explore unknown cancer therapeutic method is an urgent topic.

### Model of myosins and cadherin-mediated cell adhesion

**Figure 3:**
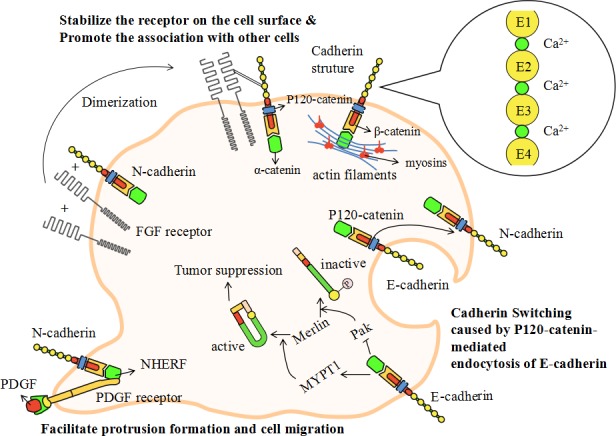
The model of cadherin-mediated cell adhesion Figure 1 has revealed the inseparable relationship between cadherins and myosins. In this picture, we focus on the detailed mechanisms and models related to cadherin-mediated cell adhesion. Cadherins are single-pass transmembrane proteins which have similar structural domains. The cadherins' extracellular domains contain five function-similarhomologous repeats (EC1-5) which are linked together by calcium ions. In the cytoplasm, cadherins connect with actin filaments by p120-catenin, β-catenin and α-catenin. Cadherin switching is a process in which tumor cells alter the physiological metabolism from expressing E-cadherin to expressing N-cadherin. This is related to the endocytosis of E-cadherin *via* the competition for the binding of p120-catenin. N-cadherin induces the dimerization of FGF (fibroblast growth factor) receptor, which directly interacts with the EC4 of N-cadherin, stabilizing the receptor on the cell surface and promoting the association with endothelial or mesenchymal cells. NHERF is a small protein serving as a scaffold to connect N-cadherin with PDGF receptor. The PDGF receptor is located at the leading edge of migrating tumor cells. The cadherin-PDGF complex can facilitate protrusion formation and cell migration. The down-regulation of merlin is a hallmark of benign tumor formation which can be modulated by cadherins. E-cadherin can efficiently activate MYPT1 and inhibit Pak. It can then phosphorylate merlin and induce an “open” inactive conformation. Active merlin is required for tumor suppression and the inactive conformation is a signal of tumorigenesis.

Earlier in this article we have illustrated the important functions of various cadherins in cell adhesion and protrusion formation. We also demonstrated the inseparable relationship between cadherins and myosins. Next, we will focus on the detailed mechanisms and models related to cadherin-mediated cell adhesion (see Figure 3).

Cadherins are single-pass transmembrane proteins which have similar structural domains. The cadherins' extracellular domain contains five function-similar homologous repeats (extracellular domain1-5 or EC1-5) which are linked together by calcium ions [201]. The cytoplasmic domain directly binds to p120-catenin and β-catenin. P120-catenin is involved in cadherin endocytosis [202], which results from the competition of different cadherins to bind to p120-catenin. β-catenin binds to the cadherins near their C-terminus and acts as an intermediate junction between cadherins and α-catenin. The intracellular actin cytoskeleton links to α-catenin and is regulated by various cadherins [201].

When tumor cells alter the physiological metabolism from expressing E-cadherin to expressing N-cadherin, they demonstrate a highly aggressive behavior. This process is called cadherin switching. Cadherin switching can significantly affect many cellular activities including those involved in cell adhesion and migration. This process is relevant for the p120-catenin-mediated endocytosis of E-cadherin [202]. It may be reasonable to suggest that N-cadherin may act to maintain steady levels of phosphorylated Rac and Cdc42, thus improving cell motility and aggressiveness [201]. Furthermore, N-cadherin induces the dimerization of FGF (fibroblast growth factor) receptor and initiates a growth-factor signal [203]. The dimeric FGF receptor directly interacts with the EC4 of N-cadherin, stabilizing the receptor on the cell surface and promoting the association with endothelial or mesenchymal cells [204]. E-cadherin also interacts with the FGF receptor but prevents FGF-dependent signaling by virtue of restricting the mobility of the receptor [205]. There is also a small protein, named NHERF, serving as a scaffold to connect β-catenin with PDGF (platelet derived growth factor) receptor. The PDGF receptor is situated at the leading edge of migrating tumor cells and its linked complex can facilitate protrusion formation and cell migration [206].

Recently, a notable difference between transformed and untransformed epithelial cells was discovered [207]. Marginal actin cytoskeletal bundles are the typical structure at the free edges of non-transformed epithelial cells. Tangential E-cadherin, located at the cell-cell joint, causes damage in these marginal actin bundles [208]. The other remaining parts of the actin bundles form two arc structures and the generated tangential stress inhibits cell protrusion formation. This process takes place not only at the cell-cell joints but at the free edges of migrating cells [207]. However, in the transformed epithelial cells, the marginal actin bundles disappear and the inner straight actin bundles remain. Once they have formed cell-cell joints, the diminished E-cadherin-based tangential stress causes the formation of overlapping cell lamellas. In this overlapping area, radial adherens junctions assemble and exert unstable functions. The stable E-cadherin-based AJs are implicated in the integrity of the epithelial cells and the maintenance of tissue stability [209]. However, the unstable radical adherens junctions are related to straight actin bundles and centripetal myosin II [210] which contributes to impaired cell adhesion and altered motility.

The above models demonstrate the detailed mechanisms of cadherin-mediated cell adhesion. Considering the inseparable relationship between myosins and cadherins, it is reasonable to assume that myosins therefore play a pivotal role in cell adhesions and migration.

### Model of myosins and cell protrusion formation

Tumor cell migration requires intracellular mechanical power to produce coordinated protrusions at the leading edge of cells. The coupling of this intracellular machinery to the extracellular environment is essential for the translocation of the cell body [45]. The formation of cell protrusions are facilitated by actin polymerization and two common protrusion structures, lamellipodia and filopodia, which display dramatically different actin reorganization and are regulated by diverse signaling mechanisms [211]. In both lamellipodia and filopodia, actin filaments take on the same polarity and elongate with the formation of the branched ends in the direction of the cell protrusions. At the roots of the actin polymers, the actin subunits are gradually released. This is responsible for the recycling of monomers and the formation of efficient protrusive power.

The lamellipodia are sheet-like cell protrusions. They are composed of a two-dimensional network of outstretched branched actin filaments [212]. The ends of actin filaments exhibit an obvious “Y” shape. Conversely, filopodia are rod-like extensions and typically contain dense bundles of about twenty actin filaments. Most protrusive cells are comprised of both filopodia and lamellipodia, thus enhancing cell migration ability [213]. The filopodia extends their roots into the lamellipodia to form a complex and cooperative structure where the lamellipodia mainly exert the mechanical power and promote locomotion and the filopodia take part in sensory reception and feedback. Moreover, it is reported that metastatic cancer cells contain more filopodia structures than non-metastatic cells [214].

A large number of proteins and molecules are involved in the formation of protrusion and play interactional roles: VASP proteins couple the ends of the actin filaments to the cell surface; capping protein and Arp2/3 regulate the capping of the actin ends; CARMIL and VASP inhibit capping and down-regulate capping proteins [211]; Arp2/3, α-actinin, myosins, fascin and filamin modulate the cross-linking of the actin filaments [215]; cofilin is required for the dissociation of the filament's roots; profilin guides the actin monomers to link to the branched ends; myosin X functions in the transportation of monomers; finally, moesin plays pivotal role in the connection of the new filament ends to the cell membrane. Other molecules have also been found related to cell protrusion formation, but their exact and detailed functions require further investigation.

**Figure 4:**
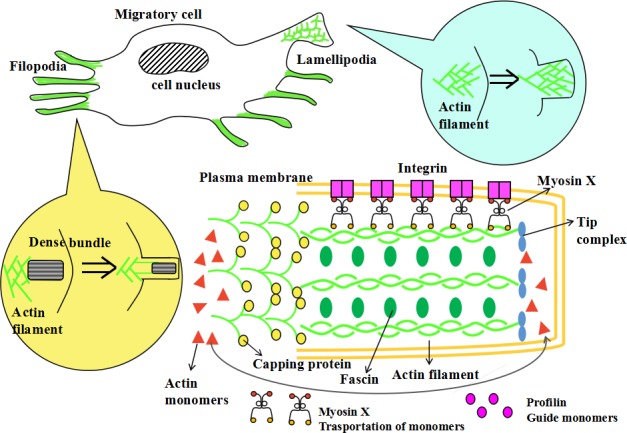
The model of myosin X and cell protrusions formation The formation of cell protrusions is facilitated by actin polymerization and various related molecules. Two common protrusion structures, lamellipodia and filopodia, have differing actin reorganizations and are regulated by diverse signaling mechanisms. The lamellipodia are a sheet-like cell protrusions. They are composed of outstretched branched actin filaments with two-dimensional network. Filopodia are a rod-like extensions and typically contain a dense bundle of about 15 to 20 actin filaments. A large quantity of proteins and molecules are involved in the formation of cell protrusions and play interactional roles. Profilin guides the actin monomers to link to the branched ends and myosin X functions in the transportation of monomers. These monomers are responsible for the recycling of actin monomers and the formation of efficient protrusive power. Capping protein regulates the capping of the actin ends. Fascin modulates the cross-linking of actin filaments and stabilizes the structures and functions of the filaments. Myosin X also has an essential functions during the formation of filopodial tips. At the surface of protrusions, integrin anchors the cell membrane to the extracellular matrix, enhancing cell adhesion. Myosin X connects integrin to the actin filaments which can then facilitate the transportation and signaling of integrins. Other molecules are also found related to cell protrusion formation, but their detailed functions requires further study.

Myosin X has essential functions during the formation of filopodia [53]. It was reported that the overexpression of myosin X could efficiently induce the formation of hundreds of filopodia per cell. However, the depletion of myosin X dramatically reduces endogenous filopodia [54]. At the surface of protrusions, integrin acts to anchor the cell membrane to the extracellular matrix. This is required for cell adhesion. Myosin X serves as a molecular linker between actin filaments and integrin and has crucial roles in the transportation and signaling of integrins. The tumor cells with mutated myosin X, lacking binding ability with integrin, decrease the formation of filopodia [55]. This suggests that the integrin-myosin X interaction is an irreplaceable factor during protrusion formation and cell motility (See Figure 4).

## A NEW CONCEPT OF “CHROMOMYOSIN”

The conventional myosin (myosin II) plays an essential role in muscle contraction and actin-dependent motility. In the cytoplasm of the cardiomyocyte, myosins convert energy from ATP hydrolysis to mechanical stress upon interaction with microfilaments. Cytoplasmic microfilaments are the binding sites and motion trails of myosins. Mutated myosins, lacking binding ability with microfilaments, aren't able to function normally. However, to date, more than seven classes of the myosin superfamily (including myosin I, II, V, VI, X, XVI and XVIII) have been discovered in the nucleus. It is well known that microfilaments don't diffusely exist in the nucleus, and just keep transient and reversible appearances [216–217], so how do these nuclear myosins exert functions effectively?

Our previous experiments show the co-localization between myosin Va and the nucleus in spermatocytes of *Eriocheir sinensis* [15]. Immunofluorescent localization analysis indicates in the presence of spermatid nuclear membrane, myosin Va enters nucleus and displays specific spatial distribution, which is similar to the distribution of KIFC1 in nucleus (named chromokinesin). Compared with the functions of chromokinesin, including chromosome condensation and spindle organization [218], we propose a hypothesis that myosin Va plays a “chromomyosin” role in nucleus. Increasing evidence suggests that other myosins participate in spindle assembly and positioning, karyokinesis, cytokinesis and other processes involved in mitosis or meiosis [14]. The mitotic spindle, which contains a dynamic array of microtubules and associated proteins, is required for normal chromosomal separation during mitosis [219]. At least three different classes of myosins have been identified to localize to the mitotic spindle and play essential roles. Depletion of myosin II is prone to impair centrosome separation and spindle assembly [220]. Myosin X is correlated with the positioning of the spindle microtubules and the maintenance of normal spindle length in *Xenopus* eggs [221]. Moreover, myosin I interacts with kinesin and efficiently improves the transportation of the spindle apparatus [222]. It is also reported that the ablation of myosin II in mouse atrial myocytes and embryonic cardiacmyocytes results in cell karyokinesis defects. This indicates the pivotal functions of myosin II in normal karyokinesis. Cytokinesis is the final stage of mitosis in which the cytoplasm is partitioned into two daughter cells. Myosin II and cytoplasmic actin are found to be located at the contractile ring and provide the motor function required for its contraction. Two structurally different myosin IIs have been identified due to their special roles during cytokinesis: Myo2p initiates the formation of the contractile ring; and Myp2p functions later to improve cytokinesis efficiency [223]. Furthermore, myosin II is also found to participate in the formation of the cleavage furrow and in chromosomal movement [224].

Furthermore, several nuclear myosins (myosin I, II, V, VI, XVI and XVIII) act with nuclear actin and regulate certain genes transcription or cell cycle [225–226]. Myosin I is regarded as the mediator of ribosomal RNA genes transcription. Actin and myosin I are both located in the specific position in the nucleolus, and the distribution of myosin I protein is closely connected to the transcription process. Meanwhile, myosin I and its related actin are responsible for the maturation of rRNA and maintenance of nucleolar structure [227]. Myosin II, as a protein first reported by scientists [228], serves as another core transcription factor to mediate the assembly of precursor complex for gene transcription. The phosphorylation or dephosphorylation of nuclear myosin light chain triggers the relative sliding between myosin II and actin, resulting in separation or connection between RNA polymerase II holoenzyme and DNA, and then decrease or increase the expression of target gene [229]. Myosin Va is found to respond the transcriptional inhibition and promote nuclear compartmentalization. The inhibition of transcription in HeLa cells caused by actinomycin D motivate myosin Va phosphorylation and cause the redistribution of myosin Va to nucleoli [230]. Nevertheless, myosin Vb is present in nucleolus, and this protein interacts with actin, RNA polymerase I and newly transcribed ribosomal RNA [231]. The roles of myosin Vb in transcription process have attracted the interest of scientists. Myosin VI is detected in the mammalian cells' nucleus, in which myosin VI co-localizes with RNA polymerase II and newly formed mRNA. On the basis of Chromatin immunoprecipitation assays, myosin VI is recruited to the promoter and activates the transcription of target genes. In the meantime, the depletion of myosin VI protein dramatically decreases the content of steady-state target mRNAs [232]. Myosin XVI is a special myosin which prominently localizes in the nucleus, especially in the nucleolus [233]. Myosin XVI interacts with stress-induced nuclear actin rods and is directed to localize at nuclear compartment. Overexpression of myosin XVI efficiently delays S-phase progression during cell cycle and prevents cell proliferation [234]. Immunolocalization results demonstrate that myosin XVIII is distributed to cytoplasm of undifferentiated myoblasts; however, after differentiation to myotubes, a part of myosin XVIII enter cell nuclei. The similar phenomena are observed in primary cardiomyocytes and skeletal muscle cells [235].

The above evidence suggests the crucial function of nucleus-related myosins. In the absence of microfilaments, myosins efficiently regulate spindle assembly, drive chromosomal movement, control mitotic dynamics and mediate gene transcription. Although we have no idea how these myosins function, we do know that these unconventional myosins play important roles in keeping normal cell division and chromosomal inheritance. Based on these viewpoints, we creatively put forward a completely new concept of “chromomyosin”. We define chromomyosin as another kind of unconventional myosin which interacts with nuclear actin, utilizes the energy from ATP hydrolysis and converts it to mechanical stress, however, unlike the other myosins, chromomyosin simply exists in the nuclear matrix and doesn't interact with cytoplasmic microfilaments. During cell mitosis and meiosis, chromomyosin participates in spindle assembly and positioning, karyokinesis, cytokinesis and other physiological processes related to cell division. This new concept impacts the traditional ideas that myosins are just the actin-based molecular motors and translocate along cytoplasmic actin microfilaments.

Nonetheless, another question is how chromomyosins translocate and walk in the nucleus. Although this problem is still controversial and has not been solved, we can still get some inspiration from existing research. In interphase nuclei, individual chromosomes are assembled within chromosome territories, which are the nuclear space of these chromosomes. These chromosome territories show nonrandom and radical positioning, which is associated with active motor proteins which includes nuclear myosin I [236]. Once actin binds to the head domain of myosin I, the complex directs myosin I's tail to link to the nuclear entity. Then myosin I conveys the nuclear entity along highly dynamic paths of nuclear actin [237]. On the other hand, during the process of myosins regulating transcription, the tail of myosin binds to the target DNA and nuclear actin is bound to polymerase. Due to the intimate interplay between myosin and actin, polymerase is led to the target DNA and activates the initiation of transcription [225].

The way how myosins enter nucleus and function as chromomyosins are still unfathomed. Some research suggests that it is the COOH-terminal tail domain, which often represents class-specific functions for various myosins, that directs chromomyosins localization and recognition on the cell nucleus [233, 238]. Other kinds of myosins, such as myosin XVI, are expressed only in nucleus and mainly locate in the nucleolus. Interestingly, some DNA replication stresses (DNA damage, deoxynucleotides insufficient or suppression of DNA polymerases) direct myosin XVI to export to the cytoplasm [233].

There are three main differences between chromokinesin and chromomyosin: First, all chromokinesins belong to two kinesin subfamilies, kinesin IV and kinesin X. These two kinesin family proteins all possess nuclear localization signals and DNA binding activity, which can explain their diffuse nuclear distribution [239]. While, the chromomyosins contain at least seven classes of myosins (including myosin I, II, V, VI, X, XVI and XVIII), which are important for spindle assembly, chromosomal movement, gene transcription, cell cycle regulation and mitotic/meiotic dynamics. Second, chromokinesins function during interphase and links to the chromosome arms during cell division. These motor proteins are responsible for chromosome segregation, normal cytokinesis and maintenance of genome stability [239]. The chromomyosins not only play a similar role with chromokinesins during interphase and cell division stage, but also take part in special genes' transcription and maturation of RNA. Third, during mitosis and meiosis, chromokinesins translocate chromosome arms along the microtubules [240]. As for chromomyosins, although they don't walk or carry cargos along the cytoplasmic microfilaments, they still interact with nuclear actin and exert specific functions; however, the polymerization status of nuclear actin is still unknown.

Chromomyosin can be described as the special and distinct myosins functioning in nucleus and directly interplaying with chromosomes. This novel concept will aid in the clarification and interpretation of the irreplaceable roles of myosins in cytokinesis and maintenance of nuclear stability. We believe that in rapidly dividing cancer cells, chromomyosin shoulders a number of indispensable functions. Studying the functions of chromomyosin during tumorigenesis may be an interesting and important topic for future research.

## CONCLUSIONS AND PERSPECTIVES

In recent years, studies have increasingly demonstrated that the myosin superfamily plays a pivotal role during tumorigenesis and cancer-related diseases. At the current research level, some questions remain ambiguous and require further investigation. For example, the actomyosin cytoskeleton has fundamental but poorly understood roles in cell adhesion and protrusion formation. The detailed functions and regulations of this remain to be clarified. In addition, the molecular mechanisms defining the different modes of cancer cell motility and migration remain unelucidated. However, one thing we can confirm is that various myosins have different but interactional functions in the formation and development of tumor cells.

Tumorigenesis is a complex and multistep process. It primarily results from the accumulation of gene mutations related to an organism's growth and development. It is now widely accepted that genetic and chromosomal instabilities are major causes of gene mutations and potential tumor progression. Subsequently, malignant cells detach from the primary tumor and enter metastasis and invasion stages. To facilitate this, a series of physiological and metabolic processes require alteration. These include the loss of cell polarity and tissue disorganization, cell protrusion formation, cell adhesion damage and the suppression of apoptosis. Seven classes of the myosin superfamily, including myosin I, II, V, VI, VII, IX and X, have been shown to participate in these processes during tumorigenesis. In addition, we have illustrated a variety of myosin-related factors, pathways and mechanisms during tumor progression, and put forward two models relevant for the tumor cell's motility and migration. Finally, a completely new concept of “chromomyosin” has been presented to interpret the special functions of myosins during karyokinesis and optimization of the myosin superfamily.

Although abundant research efforts have been conducted in the exploration of the myosins' functions during tumorigenesis, the current understanding is far from sufficient or complete. The following two aspects, in particular, require further examination. Firstly, although we have illustrated plenty of myosin-mediated factors and pathways, there are still some unknown mechanisms associated with tumorigenesis, such as myosin-related chromosomal and centrosomal amplifications, that needs to be elucidated. Future studies may focus on a panoramic view of the myosin interaction network. Secondly, the ultimate target of researching cancer-related molecules is finding the possible candidates for future cancer therapy. Accumulating evidence has proved that myosins play irreplaceable roles in the formation and development of cancer. Therefore, how to combine these myosins-related studies with medical therapy is a key next stage focus.

## ABBREVIATIONS

CDK6: cyclin-dependent kinase 6
CIN: chromosomal instability
CPI-17: protein kinase C-potentiated phosphatase inhibitor of 17 kDa
E2F4: E2F transcription factor 4
EMT: epithelial-to-mesenchymal transition
FAK: focal adhesion kinase
FGF: fibroblast growth factor
GOLPH2: Golgi phosphoprotein 2
HSPG: heparan sulfate proteoglycan
INNP5K: inositol polyphosphate-5-phosphatase K
JAK: Janus kinase
JNK: Jun kinase
MHC: myosin heavy chain
MLC: myosin regulatory light chain
MLCK: myosin light chain kinase
MMPs: matrix metalloproteinases
MRCK: myotonic dystrophy kinase-related Cdc42 binding kinase
MRTFs: myocardin-related transcription factors
MYBPH: myosin binding protein H
MYPT1: myosin phosphatase
NMHC IIA: non-muscle myosin heavy chain IIA
PAK: p21-activated kinase
PDGF: platelet derived growth factor
PIP2: phosphatidylinositol 4,5-bisphosphate
PKA: protein kinase A
PKM2: Pyruvate kinase M2
PP1: Protein Phosphatase 1
p-p38: phosphorylated p38
RhoGEF2: RhoGTP exchange factor
ROCK: RHO-associated serine/threonine kinase
Rok: Rho kinase
RTK: receptor tyrosine kinases
SDC4: syndecan-4
SRF: serum response factor
